# Sulfide Oxidation Evidences the Immediate Cellular Response to a Decrease in the Mitochondrial ATP/O_2_ Ratio

**DOI:** 10.3390/biom12030361

**Published:** 2022-02-24

**Authors:** Frédéric Bouillaud

**Affiliations:** Institut Cochin, INSERM, CNRS, Université de Paris, F75014 Paris, France; frederic.bouillaud@inserm.fr

**Keywords:** mitochondria, bioenergetics, ATP/ADP ratio, oxygen, oxygen-sensing, succinate, dioxygenase, redox state

## Abstract

The present article will not attempt to deal with sulfide *per se* as a signaling molecule but will aim to examine the consequences of sulfide oxidation by mitochondrial sulfide quinone reductase in mammalian cells. This oxidation appears first as a priority to avoid self-poisoning by endogenous sulfide and second to occur with the lowest ATP/O_2_ ratio when compared to other mitochondrial substrates. This is explained by the injection of electrons in the respiratory chain after complex I (as for succinate) and by a sulfur oxidation step implying a dioxygenase that consumes oxygen but does not contribute to mitochondrial bioenergetics. Both contribute to increase cellular oxygen consumption if sulfide is provided below its toxic level (low µM). Accordingly, if oxygen supply or respiratory chain activity becomes a limiting factor, small variations in sulfide release impact the cellular ATP/ADP ratio, a major metabolic sensor.

## 1. Introduction

Hydrogen sulfide (H_2_S) is a gas thought to have a crucial role during the early period of life on earth [1]. Nowadays, sulfide is known for its odor and toxicity for all organisms relying on mitochondrial respiration, and sulfide exposure is a concern for several human activities. Sulfide’s effect on respiration is comparable to that of cyanide (HCN/CN^−^), carbon monoxide (CO), or nitric oxide (NO). These gases are small molecules with a size comparable to molecular oxygen (O_2_) and their interaction with heme binding proteins grounds most of their toxic effects. A shared target is the mitochondrial respiratory complex IV (cytochrome oxidase) [2]. In addition, binding to hemoglobin may impact blood oxygen transport (CO). Remarkably, NO first [3,4], CO second [5,6], and H_2_S third [7] have gained the status of physiologically meaningful biological mediators [8]. Understanding the roles/effects of these three mediators and deciphering the intracellular events mediating their signaling effect is still subject to ongoing research. In this respect, the question of relationships between their signaling roles and mitochondrial toxicity deserves consideration because mitochondrial inhibition is conceivable in vivo [9,10].

The release of sulfide by sulfur-reducing bacteria in various ecosystems has the consequence that other diverse eucaryotic inhabitants have to deal with this poison [11]. Moreover, the microbiota present in the digestive tract contains sulfur-reducing bacteria, making the sulfide challenge an issue for the gut mucosa [10,12]. Finally, mammalian biochemistry entails three enzymes susceptible to release sulfide: cystathionine γ-Lyase (CSE) [13], cystathionine-ß-synthase (CBS) [14], and 3-mercaptopyruvate sulfurtransferase (3-MST) [15,16]. CSE and CBS participate in the recycling of homocysteine released by the methylation pathway involving S-Adenosyl-methionine. Exogenous and/or endogenous sulfide release therefore constitutes a threat to mitochondrial respiration. Estimations about this endogenous sulfide release in tissues resulted in values expressed in pmolH_2_S/min/mg [17]; hence, if 1 mg equals 1 microliter, equivalent to µmol H_2_S/min/L. Sulfide is toxic for mitochondrial respiration in the low micromolar range. Hence, without elimination pathways, self-poisoning by endogenous sulfide would be a matter of minutes. The more efficient means for animal cells to prevent sulfide toxicity is thought to be oxidation by a mitochondrial enzyme, the sulfide quinone (oxido)reductase (SQR or SQOR), branched to the mitochondrial respiratory chain. SQR would therefore eliminate exogenous sulfide by oxidation, and a robust SQR activity constitutes an adaptation to sulfide exposure in animals exposed to this poison [11] and in the gut epithelium [18]. In addition, SQR is found in many other cells/organs not exposed to exogenous sulfide [19]. In these places, SQR is thought to prevent toxic sulfide accumulation by normal cellular metabolism. The importance of this sulfide oxidation pathway is attested by the gravity of symptoms associated with loss of function of SQR itself [20] or of its partners in the sulfide oxidation pathway [21]. This suggests that washout of H_2_S by blood flow and elimination in the lungs is not able to guarantee a satisfying control of the concentration of this gas. With regard to the signaling role of sulfide, SQR could be understood as a controller permanently downgrading a cellular “sulfide tone”. The earliest report on the physiological role of sulfide came from neurobiology [7]. SQR expression in the brain is much lower than in other tissues [19], while sulfide generating enzymes, such as CBS and 3-MST, are present. Therefore, the brain is a place where the “endogenous sulfide tone” is likely to be more intense. An increase in the gene dosage of CBS in Down syndrome is presumed to be pathogenic [22], and an increase in sulfide-derived signaling, or bioenergetic interference, was associated with schizophrenia [23]. In both cases, the SQR pathway in other tissues is presumed normal, highlighting the restricted range for sulfide signaling and the role of endogenous sulfide-generating enzymes. Finally, environmental exposure to sulfide at subtoxic levels causes encephalopathy [24]. In addition to the extreme sensitivity of the brain to bioenergetic disturbances, it could reflect the synergistic effect of endogenous and exogenous sulfide.

The present article will not attempt to deal with sulfide as a signaling molecule but aim to summarize and revisit the conclusions of our past work and to examine how peculiar properties of the mitochondrial sulfide oxidation pathway could shed light on cellular bioenergetics and regulations.

## 2. Chemistry

H_2_S upon dissolution in water dissociates into the anions HS^−^ and S^2−^ whose relative proportions depend on the pH of the solution. At a physiological pH, the ratio H_2_S/HS^−^ is approaching 1/2 and S^2−^ is negligible. In the present text, sulfide means the sum of gas H_2_S and of these anions. The part of sulfide present as H_2_S in solution equilibrates with the surrounding atmosphere this causes significant sulfide loss, and eventually disappearance, in any experiment at physiological (or acid) pH with an air/liquid interface.

**Figure 1:**
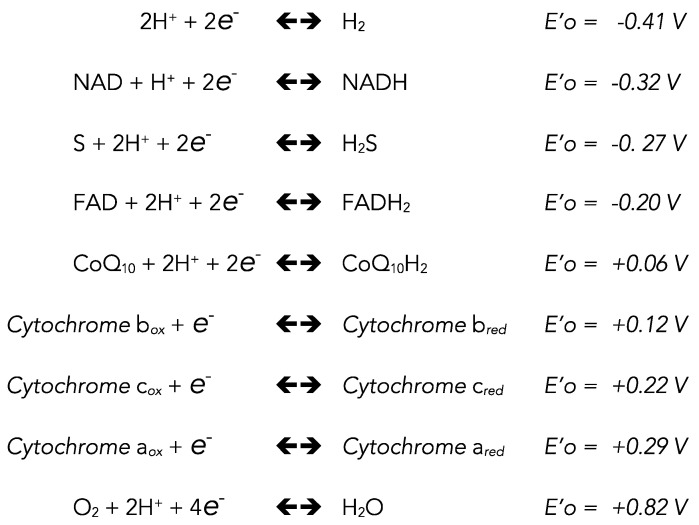
Apparent standard reduction potentials.

The sulfur is on the same column as oxygen and selenium (chalcogens). A first possibility for sulfur to fill its outer electronic layer is to gain two electrons. This is the case with H_2_S, the comparison could easily be made with H_2_O. However, while the redox potential for oxygen reduction by hydrogen is strongly positive (+0.8 V) with the result that the breakdown of H_2_O needs a considerable amount of energy (photosynthesis), the redox potential for the reaction between sulfur and hydrogen (−0.27 V) is in the same range as well-known biological redox intermediates, such as NAD and FAD (Figure 1).

**Figure 2:**
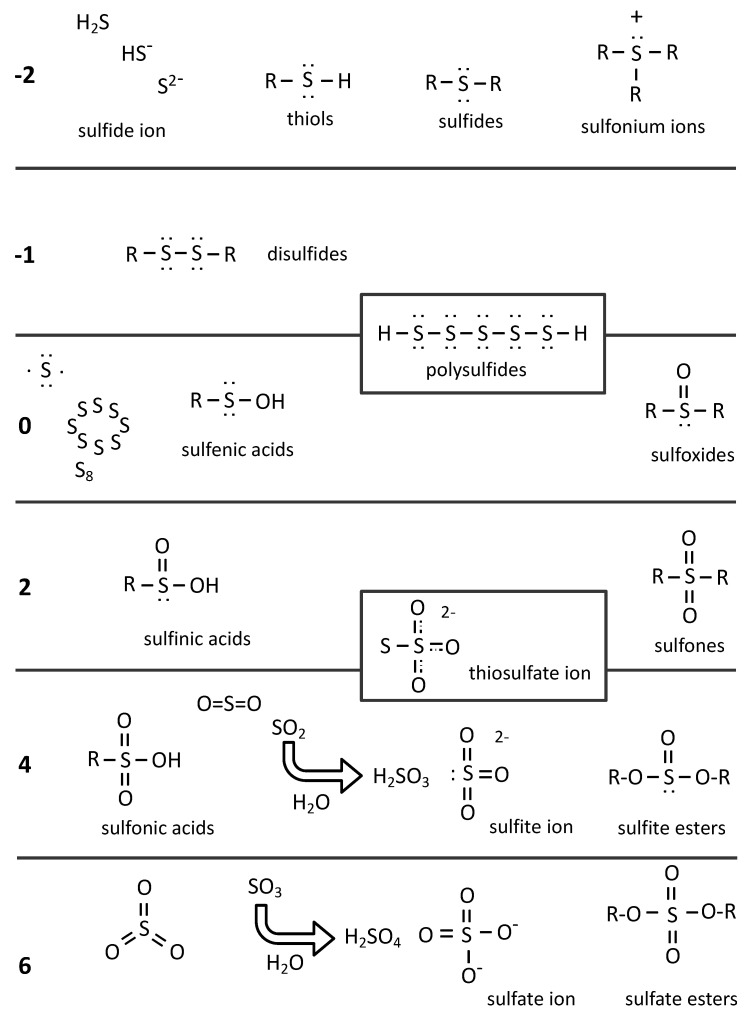
Oxidation state of sulfur compounds. Oxidation states of sulfur are indicated on the left by bold numbers. For the zero state, the outer electronic layer of sulfur is schematized top left. An example of polysulfide is shown boxed over columns −1 and zero; the two sulfur atoms at the edges are at state −1 and in the center at state 0. For states 4 and 6, we show the hydration products of sulfur dioxide and sulfur trioxide, leading to the formation of sulfite and sulfate ions, respectively. Thiosulfate is boxed between states 2 and 4. This figure is inspired by Chemistry Libretexts Thiols and sulfides contributed by William Reusch: https://chem.libretexts.org/Bookshelves/Organic_Chemistry/Supplemental_Modules_(Organic_Chemistry)/Thiols_and_Sulfides accessed on 7 January 2022.

Sulfur in the native state is in the zero-oxidation state (S_0_); native sulfur is mostly found with eight atoms linked together forming a ring (octasulfur, S_8_). In H_2_S (sulfide), the oxidation state of sulfur is −2. This same value is considered to apply for the sulfur atom engaged in C-SH bonds, such as those present in cysteine. Sulfur can also associate with atoms attracting more vigorous electrons than it does, such as oxygen. Oxidation of sulfur by dioxygen leads to SO_2_ (a gas); its reaction with water forms sulfurous acid (H_2_SO_3_) that dissociates into protons and a sulfite ion, SO_3_^2−^. The oxidation state of sulfur in sulfite is +4; further oxidation leads to sulfate (SO_4_^2−^), in which the sulfur oxidation state is +6. Then, the oxidation state of sulfur covers a wide range from −2 to +6 (Figure 2).

## 3. Oxidation of Sulfide by SQR

Sulfide Quinone Reductase (SQR) is an enzyme originally found in several types of bacteria that allows H_2_S as an energy substrate; hence, SQR yields protons and electrons from sulfide to the bacterial “bioenergetic machinery” at the level of the quinone present in the membrane [25,26]. The oxidation of sulfide by SQR could release sulfur in two oxidation states, −1 or zero. In polysulfides of a general formula, HS-S_n_-SH, the two sulfur atoms on the edges are at the oxidation state −1, while the n sulfur atoms in the center are at the zero state. When disulfide (HSSH) is considered, the oxidation state is −1 for both atoms. The consequence is that the ratio between sulfide use and reduction of quinone (Q to QH_2_) by SQR could theoretically vary from 2 H_2_S per quinone (release of disulfide) to 1 if one H_2_S reduces one quinone with release of sulfur (S_0_) and for example a ratio of 1.1 sulfide per quinone has been reported with the SQR from *Rhodobacter capsulatus* [26], it is equivalent to formation of H_2_S_11_ and means therefore that reduction in S_0_ dominates. The catalytic cycle of mammalian SQR differs from that of the bacterial enzyme [20]. However, it remains that the sulfur atom of sulfide (oxidation state −2) is expected to have increased its oxidation state to −1 or zero.

### 3.1. Evidencing Mitochondrial SQR Activity

**Figure 3:**
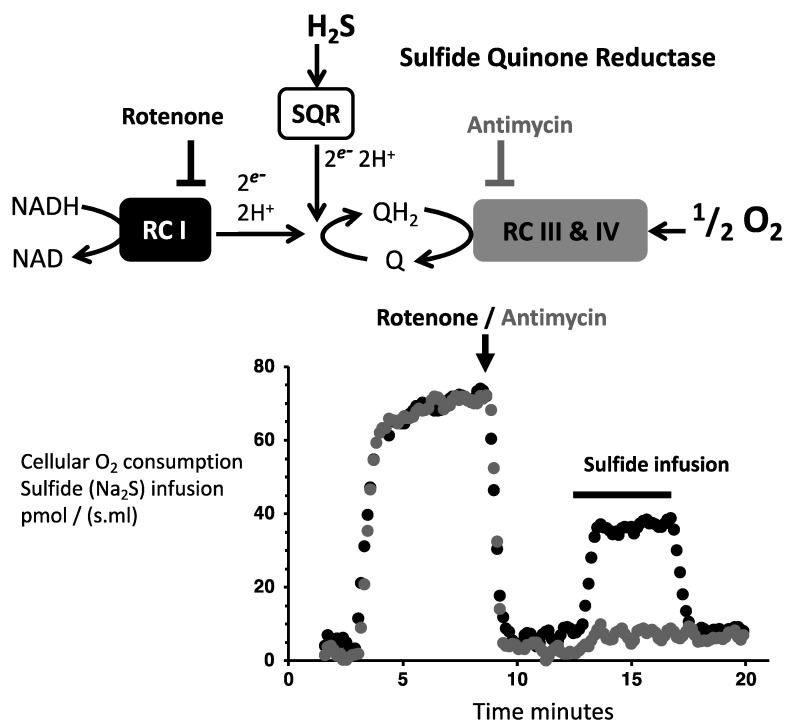
Mitochondrial Sulfide Quinone Reductase activity. **Top**: Branching of SQR on the mitochondrial respiratory chain the mitochondrial complexes I (RC I) and III and IV (RC III & IV) are shown as boxes. **Bottom**: Experiment with inhibitors to demonstrate sulfide quinone reductase activity in the mitochondrial respiratory chain. The X-axis time in minutes, the *Y*-axis rates of cellular oxygen consumption (CHO cells ≈ 10^6^ cells/mL) or sulfide infusion, and the oxygen consumption rate were followed simultaneously in two chambers. Experiments started by closure of the chambers (before 5 min), and after recording endogenous cellular respiration (from 5 to 8 min approximately), the inhibitors of respiratory complex I (rotenone) or III (antimycin) were added (black/gray trace respectively). Then, the same sulfide infusion was settled in the two chambers for the period of time indicated by the black horizontal bar. Oxygen consumption increases synchronously with infusion in the presence of rotenone, while it remains low in the presence of antimycin. Remember that the Y ordinate (45) for the horizontal black bar refers to the sulfide flux, which could be directly compared to the oxygen consumption rate it caused (here roughly 30); hence, in this experiment O_2_/H_2_S = 0.67. Note that colored versions of Figures 3–10 are provided in the Supplementary Material.

The assessment of the SQR activity could be made easily by providing sulfide to cells or mitochondria in which no substrate other than sulfide could be used. This applies to isolated mitochondria or to cells in which endogenous respiration is inhibited by a complex I inhibitor, such as rotenone. This is because all cellular quantitatively relevant metabolic oxidation pathways (glucose, fatty acids, proteins) depend on the NAD/NADH redox cycle that requires complex I activity and are therefore impaired when rotenone is present (Figure 3). SQR activity results in the reduction of quinone that is re-oxidized by mitochondrial respiratory complex III to reduce cytochrome c. The reduced cytochrome-c is oxidized by mitochondrial respiratory complex IV to reduce oxygen in water (RC III & IV in Figure 3). This means 1/2O_2_ per quinone and therefore with regard to the mitochondrial respiratory chain, one would expect O_2_/sulfide ratio values rank from 0.25 to 0.5 with sulfur atom released at oxidation state −1 or zero, respectively.

Oxidation of sulfide (SQR activity) is therefore indirectly quantified by the increase it causes to oxygen consumption in the presence of rotenone. Increase in cellular oxygen consumption immediately follows initiation of sulfide infusion because gas diffusion ensures fast transfer of extracellular sulfide to mitochondrial SQR. The inhibition of complex III or IV makes the overall reaction impossible. Therefore, the complex III inhibitor antimycin prevents a fast increase in cellular oxygen consumption caused by sulfide and authenticates the release of electrons from sulfide to quinone. This procedure allowed us to demonstrate that nanomolar concentrations of sulfide stimulate SQR [27]. Notably, sulfide at micromolar concentration binds reversibly to complex IV and inhibits its activity. Therefore, sulfide inhibits its own oxidation, generating positive feedback schemes for inhibition establishment and release [28,29]. For this reason, the addition of enough sulfide to feed respiration for minutes is not feasible, and low µM sulfide additions have to be considered. Then, only transient increases in cellular (mitochondrial) oxygen consumption are observed with a return to the background rate when sulfide is exhausted [28,30]. Another option is to use sulfide infusions at rates below the maximal possible SQR activity. This procedure generates steady states with a sustained increase in oxygen consumption strictly synchronized with the sulfide infusion (Figure 3, bottom). If sulfide infusion exceeds the oxidation rate, accumulation of sulfide has the consequence that increase in oxygen consumption persists after the infusion ceases or worse that inhibition occurs during infusion. This criterion could be used to obtain a direct estimation of the cellular maximal sulfide oxidation capacity (SQR activity) [18,19].

### 3.2. Oxygen (O_2_) to Sulfide Ratio

**Figure 4:**
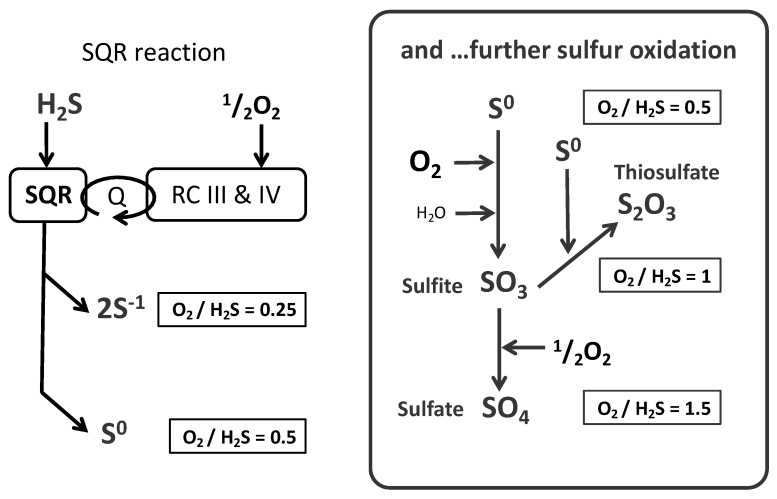
Oxygen consumption and sulfide oxidation. SQR yields two electrons and protons to the respiratory chain (RC III & IV), with the oxidation of one sulfide into S^0^ or to two sulfides releasing two S^−1^. On the right (boxed), subsequent sulfur oxidation steps from S^0^. The respective O_2_/H_2_S ratios are indicated near the reaction products. Note that the oxygen consumption from sulfite to sulfate is explained by the activity of the respiratory chain.

It is a reasonable assumption that under these conditions of steady state, the sulfide oxidation rate equals the sulfide infusion rate (Figure 3). Therefore, the ratio between the increase in oxygen consumption and the sulfide infusion rate provides an estimation of the O_2_/sulfide ratio. The mean value approached 0.8 [18,19,30] and then clearly above the range 0.25 to 0.5 predicted to result from quinone re-oxidation. This was not unexpected since sulfur oxidation was thought to continue after the SQR reaction [18]. The possible oxidation products resulting from the combination of oxygen and sulfur are shown (Figure 4 right box).

When respiratory chain and sulfur oxidation are summed, the different possible oxidation schemes give O_2_/Sulfide ratio with values of 1 (two sulfides and two O_2_ form thiosulfate S_2_O_3_^2−^), 1.5 (SO_3_^2−^) and 2 (sulfate SO_4_^2−^), which are significantly higher than the experimental value of 0.8. Hence, while the O_2_/sulfide ratio evidenced the occurrence of further oxidation of the sulfur product of SQR, it did not fit with an oxidation scheme, as shown here (Figure 4). Possible explanations for the discrepancy between the experimental value and any of those expected from a single reaction scheme included: (i) experimental bias resulting from a solution with partially impure/degraded sulfide, (ii) an oxidation scheme different from those presented above, or (iii) a combination between a direct release of the SQR product (polysulfide/sulfur) and post SQR sulfur oxidation [18], which depending on their relative contributions, would result in a global value between 0.25 and 2.

### 3.3. Infusions in Presence/Absence of Endogenous Respiration

The existence of intracellular enzymatic steps releasing sulfide indicates that cells are under the constraint of a sustained endogenous sulfide flux. Then, to reproduce conditions closer to normal cellular physiology, the effect of sulfide infusion was compared on the same cellular suspension in the presence or absence of rotenone. In the absence of rotenone, sulfide oxidation took place in the presence of “endogenous respiration”, a term which will be used hereafter to mention this use of normal (non-sulfide) mitochondrial substrates. Endogenous respiration did not interfere with the rate of sulfide oxidation since an increase in oxygen consumption rate was observed in both cases, and it remained strictly coincident with the sulfide infusion period [19,27]. Endogenous cellular respiration and sulfide oxidation share the same electron transfer pathway between quinone and oxygen (Figure 3). Then, sulfide oxidation takes place irrespective of the presence/absence of pre-existing existing electron transfer in this common pathway and therefore would sum up with endogenous respiration or take precedence over it.

Further information came from the value of the increase in O_2_ consumption over endogenous respiration. The O_2_/sulfide ratio for this increase was lower in the presence of endogenous respiration, with a value around 0.5. Although this was not demonstrated, it was assumed that the overall sulfide oxidation reaction was the same in the presence/absence of rotenone. Then sulfide oxidation does not simply sum up with endogenous respiration because this would result in identical results for the O_2_/sulfide ratio in the presence/absence of rotenone. This lower O_2_/sulfide ratio in the absence of rotenone was thought to reflect the interference between sulfide oxidation and endogenous respiration. Then the difference in O_2_/sulfide ratio would be easily explained if electrons from SQR replace those coming from other substrates. NADH reoxidation, hence complex I activity, is the largest contributor for electron injection in the respiratory chain: 80% or more for full oxidation of glucose and 67% with fatty acids. In other words, SQR takes precedence over complex I to reduce quinone in the mitochondrial respiratory chain, and initiation of sulfide oxidation impacts immediately on the reduction state of intracellular NADH [19].

There are two consequences associated with this observation. Firstly, it raises the possibility that the incoming sulfide flux could increase up to the point at which all electrons injected in the respiratory chain come from sulfide (if SQR activity is high enough). Actually, adaptation of the colonic epithelial cells makes even more possible since SQR could reverse electron transfer in complex I, then two opposite electron fluxes starting from the reduced quinone sum up to evacuate the electrons provided by SQR [19,27].

Secondly, it provides indications on the sulfide oxidation process since in the absence of rotenone, the contribution of the post-SQR reaction(s) to oxygen consumption is enhanced. The assumption that “electrons from SQR replace electrons from other sources” suggests that the overall electron transfer by respiratory chain to oxygen remains the same. If so, the increase in oxygen consumption in the presence of endogenous respiration reflected exactly the contribution of the post-SQR oxidation pathway [19]. Since the same, hence equally impure, sulfide infusion was infused, the relative variation of the O_2_/sulfide ratio should not be influenced by purity. While the values of 0.8 and 0.5 are subject to caution, the O_2_/sulfide ratio in the presence of rotenone is thought to really be about 1.5 times that observed in its absence, which would mean that the oxidation of the SQR sulfur product consumed approximately two times more oxygen than that the reoxidation of the quinone. This collides with the share observed with thiosulfate formation, for which contribution of respiratory chain and of sulfur oxidation pathway are equal. It would fit with sulfite formation but means a factor close to two between theory (1.5) and experimental value (0.8) for the stoichiometry O_2_/H_2_S. A significant polysulfide release by SQR is unlikely because it lowers overall stoichiometry but increases the relative contribution of respiratory chain to O_2_ consumption. Actually, in order to match with experimental values, a theoretical ratio of 0.75 O_2_/sulfide was proposed with 0.5 for post-SQR sulfur oxidation and 0.25 for respiratory chain [19]. Hence, disulfide would be the intermediate before further oxidation. Then, to comply with this theoretical value, the final oxidation product would result from the reaction of disulfide (H_2_S_2_) with one O_2_, but formula H_2_S_2_O_2_ is different from thiosulfate (H_2_S_2_O_3_). We suggest hereafter that a wider look considering cellular physiological constraints resolves this “chemical contradiction” and furthermore highlights how a marginal sulfide releasing rate may alter intracellular signaling by interference with cellular bioenergetics.

## 4. A Model for Sulfide Oxidation with Preservation of Cellular ATP Turnover

**Figure 5:**
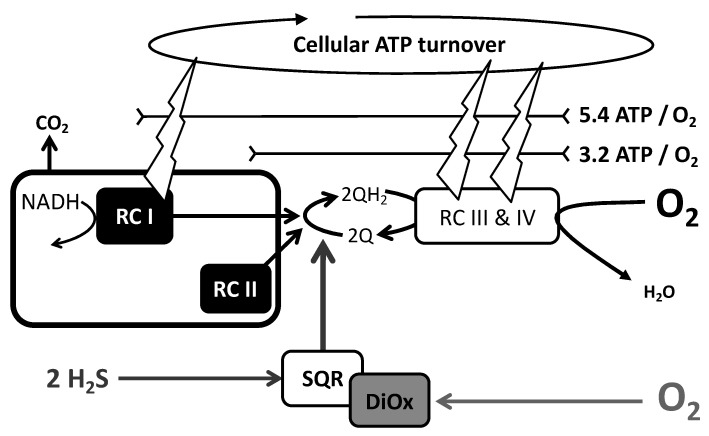
Cellular ATP turnover model. SQR and other redox enzymes, such as respiratory complexes I (RC I) and II (RC II), reduce the common redox intermediate quinone (Q/QH_2_) whose reoxidation by respiratory complexes III and IV yields the reduction of oxygen (O_2_) in water. RCI, III, and IV energize the mitochondrial membrane (symbolized as sparks), which ensures ADP phosphorylation into ATP by complex V (not shown) and therefore feed the cellular ATP turnover. RC I and RC II are linked to carbon oxidation schematized as a black box releasing CO_2_. SQR activity is associated with further oxygen consumption caused by dioxygenase (DiOx); here, thiosulfate is considered the final product (2 H_2_S and 2O_2_). ATP regeneration depends on proton pumping by complexes I, III, and IV; it results that the ratio ATP/O_2_ has different values according to the electron donor NADH (complex I) or FADH2/FMNH2 (complex II or SQR). A mixed contribution of carbon and sulfide oxidation will be considered with regard to the contribution to cellular ATP turnover, taking into account the constraint that sulfide oxidation is an absolute priority.

Living cells have high energy requirements; most of the energy comes from the hydrolysis of the ATP molecule into ADP, which has to be compensated by continuous regeneration of ATP, which essentially relies on mitochondrial respiration. Hence, cellular energy needs are met by intense ATP turnover. Then, while the discussion above referred to the electron flux caused by sulfide oxidation, we will now consider the contribution of mitochondrial sulfide oxidation to this cellular ATP turnover. The ATP/O_2_ ratio for sulfide is low because the two electrons from sulfide arrive at the level of quinone, hence with an ATP/O_2_ value of 3.2 compared to 5.4 when electrons come from NADH/complex I (Figure 5). This same value of 3.2 is observed with oxidation of succinate into fumarate catalyzed by the succinate dehydrogenase (SDH), which is complex II of the mitochondrial respiratory chain. This value (3.2) also applies to other enzymes using FAD/FMN to link the metabolic oxidation step to quinone reduction. These values of ATP/O_2_ ratios are maximal and theoretical because they depend on the fact that 100% of protons contribute to ATP synthesis, hence in absence of other pathways for proton return through the mitochondrial inner membrane usually mentioned as “proton leak”. This proton leak includes non-productive proton return (partial uncoupling) but also coupled ion transport, for example export of potassium or calcium ions ensuring mitochondrial ionic homeostasis (Ca^++^) and volume control (K^+^). Assuming a leak of 20% of protons would lower the ATP/O_2_ values indicated above to 2.56 and 4.32, respectively. Their relative magnitudes are unchanged, and considering or not the leak has no consequences for comparison of the relative efficiencies in ATP/O_2_ for the different oxidative schemes in the mitochondrial respiratory chain. Therefore, because there is no consensus value for the leak, the theoretical values (3.2, 5.4) will be considered hereafter.

**Figure 6:**
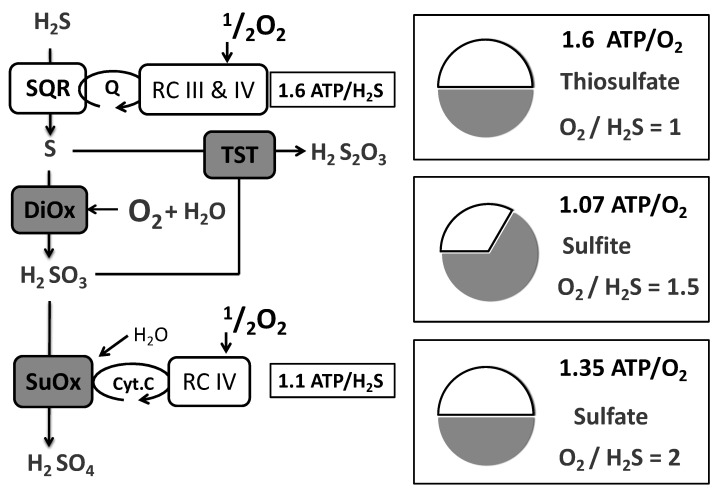
O_2_/sulfide ratio and ATP/O_2_ ratio. (**Left**) sulfide oxidation pathway SQR releases S_0_ that can be further oxidized by a dioxygenase (DiOx) presumably ETHE1, the resulting molecule H_2_SO_3_ (sulfite) could be subject to the addition of another S^0^ atom (from a second SQR reaction) by a sulfur transferase (TST), presumably rhodanese, to form thiosulfate (H_2_S_2_O_3_) or to further oxidation into sulfate (H_2_SO_4_) by sulfite oxidase (SuOx) that yields an electron to complex IV of the respiratory chain. When thiosulfate or sulfite are the final products, ATP production results from electron transfer from quinone to oxygen, hence 1.6 ATP per H_2_S engaged in the oxidation pathway. Thiosulfate formation consumes two sulfide and oxygen molecules, hence 3.2 ATP/2O_2_. If sulfite is the final product, one sulfide and 1.5 oxygen molecules are used, hence 1.6 ATP/1.5O_2_. When sulfate is the final product, sulfite oxidation with electron transfer from cytochrome-c to oxygen results in 1.1 ATP per sulfite, hence per H_2_S engaged in the oxidation pathway. The sum is therefore 2.7 ATP (1.6 + 1.1) and two oxygen molecules, hence 2.7 ATP/2 O_2_. (**Right**) resulting O_2_/H_2_S and ATP/O_2_ ratios for the different sulfide oxidation products. The graphics show the relative contribution of respiratory chain (empty) and sulfur oxidation (gray) to oxygen consumption.

Moreover, in contrast with these enzymes, further steps of sulfur oxidation lower the ATP/O_2_ ratio even more (Figure 6). If thiosulfate is the final product, ATP/O_2_ for sulfide oxidation is lowered to 1.6 and the pathway from sulfide to sulfate (the physiological form for elimination of sulfur) predicts a value of 1.35. The O_2_/H_2_S ratio quantifies the amount of oxygen required for detoxication of sulfide; release of thiosulfate maximizes oxygen efficiency, while that of sulfate is the worst situation. A large contribution of sulfite release is unlikely: sulfite is a substrate for the two enzymes TST and SuOx (Figure 6), it is a toxic compound [31,32], a bad option with regard to O_2_/H_2_S ratio and the worst with regard to ATP/O_2_ (Figure 6).

**Figure 7:**
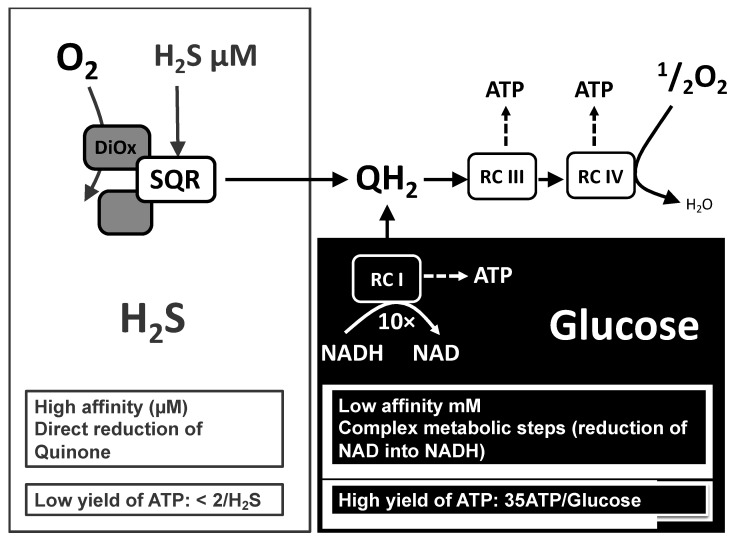
Comparison of the two energy substrates glucose and sulfide. Physiological concentrations of glucose in the external medium are maintained in the millimolar range to ensure support of cellular metabolism. In contrast, the Km for rat liver mitochondrial SQR is 2 µM and evidencing sulfide oxidation in the nanomolar range is feasible (Supplementary Figure S1). Glucose oxidation into carbon dioxide implies ten metabolic steps reducing NAD into NADH, the substrate for mitochondrial complex I. Other metabolic steps include substrate-linked ATP generation steps and reduction of quinone by complex II (not shown). Altogether, it results in a large difference between the yields expressed in ATP generated per substrate oxidized; a similar conclusion applies if oxygen consumption is considered (Figure 6 and text).

As previously mentioned, sulfide present in the external medium becomes available to mitochondrial oxidation within a short time, and sulfide oxidation takes precedence over other mitochondrial substrates. Hence, at the cellular level, sulfide infusion causes an immediate switch from “normal cellular energy substrates” to sulfide, and therefore the ATP/O_2_ of cellular respiration is immediately lowered. Glucose will be considered an example of the “normal cellular energy substrate”. Complete oxidation of glucose is predicted to yield 35 ATP and to consume 6 molecules of oxygen; therefore, the ATP/O_2_ is therefore 35/6 = 5.7, which contrasts sharply with the low values shown above for sulfide. There are other differences, and a comparison of these two cellular energy substrates is summarized in Figure 7.

**Figure 8:**
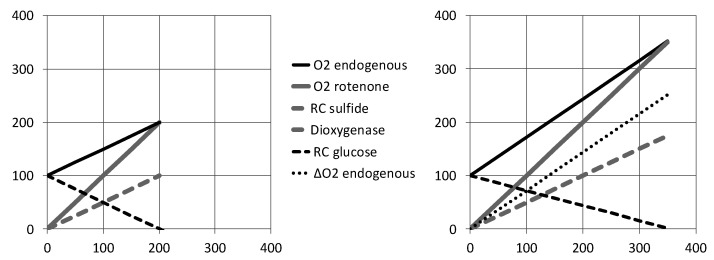
Impact of sulfide oxidation on cellular oxygen consumption. The X-axis sulfide infusion rate, Y-axis oxygen consumption rate represented with the same units: moles/(time × volume). Endogenous respiration is supposed to be fed by glucose with a value of 100 at the origin of the X-axis (sulfide infusion = 0). Sulfide infusion is then increased to the point where all electrons in the respiratory chain come from sulfide, hence when oxygen consumption caused by glucose oxidation is lowered to zero: “RC glucose” (dashed black line). The oxygen consumption rate for sulfide oxidation into thiosulfate is the sum of respiratory chain “RC sulfide” and of “Dioxygenase” contribution that are equal (dashed gray line). Their sum therefore predicts the increase in O_2_ consumption rate if endogenous cellular respiration is poisoned by rotenone: “O_2_ rotenone” (solid gray line). The sum “RC sulfide + Dioxygenase + RC glucose” predicts the cellular oxygen consumption rate in the presence of endogenous respiration: “O_2_ endogenous” (solid black line). The impact of sulfide oxidation on glucose oxidation is shown with two different models: (**Left**) if electrons from SQR simply replace those coming from carbon oxidation. (**Right**) If a constant ATP turnover is maintained, which implies correction for the different ATP/O_2_ yields of glucose (5.7) and sulfide (1.6), the dotted black line, “∆O_2_ endogenous,” highlights that with this model, the increase in oxygen consumption caused by sulfide in the presence of endogenous respiration represents more than the contribution of the dioxygenase.

We will consider the hypothesis that cellular sulfide oxidation releases thiosulfate. Then, the contribution of sulfide oxidation to ATP turnover is considered from zero to the point it covers 100% of electron transfer in the respiratory chain (Figure 8). On the left, a constant electron flux in the respiratory chain is considered as in [19]; dioxygenase and SQR contribute equally to oxygen consumption, and in the presence of endogenous respiration (red line), the maximal possible flux of sulfide causes a two times increase in oxygen consumption (Y max = 200). On the right side, the ATP turnover rate is supposed to be constant. The endogenous O_2_ consumption rate of 100 predicts an ATP turnover rate of 570 if glucose is the cellular energy substrate. To reach this same value with oxidation of sulfide into thiosulfate, the O_2_ consumption rate would be 570/1.6 = 356 O_2_ with use of the same amount of sulfide (O_2_/sulfide = 1). Hence, if SQR activity is high enough to support 100% of mitochondrial ATP production, the maximal tolerated sulfide infusion rate and oxygen consumption might be as high as 3.56 times the endogenous O_2_ consumption rate observed in the absence of sulfide (Figure 8 right, solid black line). Actually, to compensate for the lower ATP yield of sulfide oxidation, the electron transfer in the respiratory chain is increased (the sum of the Y axis for the gray and black dotted lines exceeds 100). As a consequence, the increase in oxygen consumption over the endogenous rate caused by sulfide infusion represents more than the contribution of the dioxygenase alone, and the ratio between oxygen consumption increase in the presence of rotenone (solid gray line) and in its absence (dotted black line) is predicted to be 1.4. Then, a thiosulfate release with preservation of the ATP turnover rate fits rather well with the experimental value close to 1.5 [19].

### 4.1. Limitation by Oxygen Supply: A Marginal Sulfide Flux Impacts on Cellular Bioenergetics

**Figure 9:**
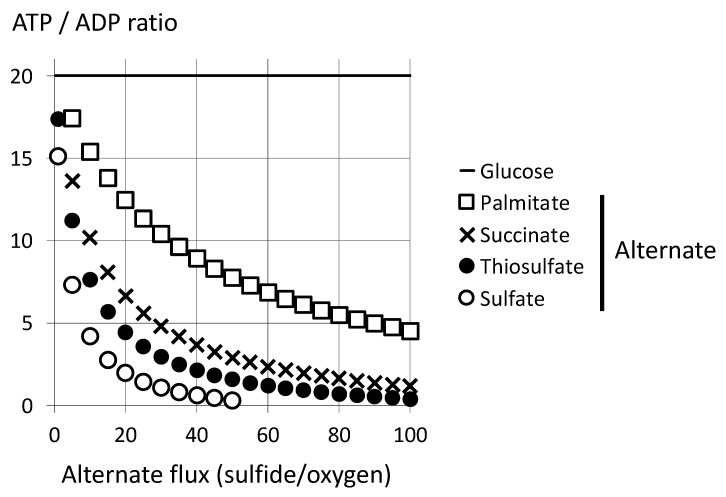
Impact of sulfide oxidation under oxygen limitation. The model states that (1) oxygen supply is the limiting factor; a fixed oxygen amount is used. (2) With this amount of oxygen, the endogenous respiratory rate with the reference substrate (glucose) would regenerate once each cellular ATP molecule (hence for a time period allowing one cycle of turnover of intracellular ATP content). (3) The decrease in ATP regeneration rate caused by intervention of alternate pathways of oxidation results in an equal increase in ADP (no decrease in cellular ATP consumption). For calculation, we consider a reference cycle with 100 oxygen (O_2_) and an ATP/ADP ratio with a value set to 20, hence 570 ATP and 28.5 ADP. The X axis represents the number of oxygen molecules used for oxidation of the alternate substrates: palmitate (ATP/O_2_ = 5) or succinate (SDH reaction only ATP/O_2_ = 3.2). With regard to the alternate substrate sulfide, the X axis represents the number of sulfide molecules consumed, and oxidation into sulfate or thiosulfate only is considered. Sulfate formation consumes two oxygen per sulfide, and therefore the corresponding curve ends with X = 50. The Y axis represents the new cellular ATP/ADP ratio after one cycle and for increasing contribution of the alternate pathways from zero to 100 with increment of five. In addition, the values are shown for X = 1 if the substrate is sulfide. The horizontal black line represents the reference value (20) for the steady state of ATP/ADP ratio with endogenous respiration oxidizing glucose.

The previous analysis was based on a maintained ATP turnover guaranteed by an increase in cellular oxygen consumption as sulfide infusion increased (Figure 8). This requires that neither oxygen supply nor activities of the respiratory chain (complexes III and IV) become limiting. We shall refer first to oxygen limitation because it is thought to be the most physiologically relevant issue and it has the more dramatic consequences. Accordingly, if a fixed oxygen supply (100) is considered, the figures with sulfide depend on the oxidation scheme (Figure 6). If the final product is thiosulfate, 100 sulfides are consumed and 160 ATP are generated. If sulfate is the end product, 50 sulfides are consumed and 135 ATP are produced, to be compared with the reference value of 570 ATP with glucose. This predicts ample consequences on the ATP generation rate from a shift from glucose to sulfide under conditions of oxygen limitation. Accordingly, a simple model could be used to evaluate the impact of the occurrence of sulfide oxidation on the cellular ATP/ADP ratio (Figure 9).

Under conditions of limitation by oxygen supply, the relative impact on the ATP/ADP ratio is highest for the lowest rates of sulfide oxidation, and the first data point for sulfide oxidation (X = 1) means a sulfide flux representing 1% of the oxygen consumption rate. If the final oxidation product is sulfate, the impact becomes larger than with thiosulfate. The high sensitivity of the ATP/ADP ratio is explained by the high reference value for the steady state under glucose oxidation (20), and lower reference values result in milder effects. However, twenty as the reference value appears justified: firstly, a high ATP/ADP ratio is a condition for cell survival, and secondly, although they were possibly underestimated because hydrolysis of some ATP could never be fully excluded, experimental values approached this value [33]. Then, variation of sulfide flux in the range of a percent of the total oxidative metabolism may impact significantly on the ATP/ADP ratio, a major metabolic sensor, and therefore lead to subsequent signaling. This bioenergetics-based sulfide signaling is a direct result of sulfide disappearance, and this would preclude a direct evaluation of the intensity of this sulfide-derived signaling from sulfide presence.

These observations are expected to apply to any factor lowering the ATP/O_2_ ratio under conditions of limitation by oxygen supply. This could be physiological substrates or metabolic troublemakers (uncouplers, redox active compounds). To illustrate this, the influence of the shift from glucose to sulfide is compared with that from glucose to palmitic acid (ATP/O_2_ = 4.9). Accordingly, the same sulfide flux would be five or ten times more efficient than a shift to fatty acid oxidation: consider in Figure 9 the first sulfide symbols (circles, with X = 1% of oxygen flux) and first or second squares (5% and 10% of oxygen consumption to palmitate, respectively). The consequences of increased contribution of succinate oxidation are shown as well (Figure 9), and although the difference is less marked than with palmitate, sulfide influx impacts the ATP/ADP ratio more than the increase in the complex II reaction. Notably, the relative efficiencies between sulfide and other substrates are unchanged with calculations using lower values for the reference ATP/ADP ratio (not shown). Figure 9 refers to the sulfide flux compared to the oxygen flux for the oxidation of other substrates. This inconsistency is justified by the fact that sulfide flux and the oxygen consumption rate were expressed in the same units; the first would quantitate the “sulfide challenge” and the second the intensity of cellular energy metabolism. This highlights that a sulfide flux of a modest extent with regard to oxidative metabolism would have consequences. The comparison with consistent X units (oxygen diversion to substrates alternate to glucose) maintains a roughly five times higher impact for sulfide oxidation compared to lipid oxidation (Supplementary Figure S2). The curves reflect ATP/O_2_ ratios making sulfate and thiosulfate curves much closer.

Of course, the quantitative importance of lipids would mean effects of higher amplitude than for physiological sulfide fluxes. However, the shift from glucose to lipid oxidation requires (coincides with) the middle/long term transition between fed state and starvation, leaving more time for adaptations aiming to compensate for the degradation in the ATP/O_2_ ratio. In contrast, variations in sulfide flux are conceivable in the short term and would quickly result in “bioenergetic signaling”. Of course, a marginal decrease in the sulfide endogenous/exogenous production rate would have the opposite consequences and would improve the cellular ATP/O_2_ hence efficiency of oxygen.

### 4.2. Limitation by Respiratory Chain Activity

Examination of the limiting factors for cellular respiration in relation to pathologies evidenced a role for what is called “the respiratory reserve”. This term means the maximal possible rate of electron transfer in the respiratory chain. If it becomes the limiting factor for cellular ATP turnover, cells become extremely sensitive to challenges, and improving oxygen supply would be useless. SQR and other quinone reducing pathways converge (Figure 5), and therefore the maximal activities of complex III or IV appear the more likely to be limiting. Such a limitation may reflect that the sum of NO, CO, and H_2_S steady state concentrations partially inhibit mitochondrial complex IV.

**Figure 10:**
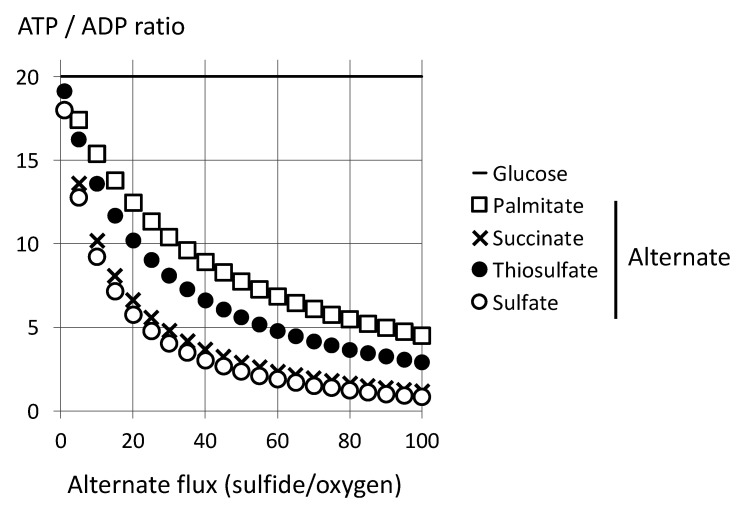
Impact of sulfide oxidation under respiratory chain limitation. The model states that the number of reactions in the mitochondrial respiratory chain is the limiting factor. Then the calculation considers 100 reactions in respiratory complexes III and IV (thiosulfate) or 50 in complex III and 100 in complex IV (sulfate see Figure 6). Rest of the legend as above. With regard to Figure 9, it modifies Y values for sulfide curves only; another consequence is the increase of the maximal X value (100) for oxidation of sulfide into sulfate.

In these conditions, the increase in oxygen consumption brought by dioxygenase is of no importance, and this mitigates the impact of sulfide oxidation on the ATP/ADP ratio (Figure 10). In contrast, the consequences of others (such as fatty acid or succinate oxidation) are unchanged. Then, in this model, succinate oxidation by complex II equals the effect of oxidation of sulfide into sulfate and outperforms sulfide oxidation in thiosulfate.

**Figure 11:**
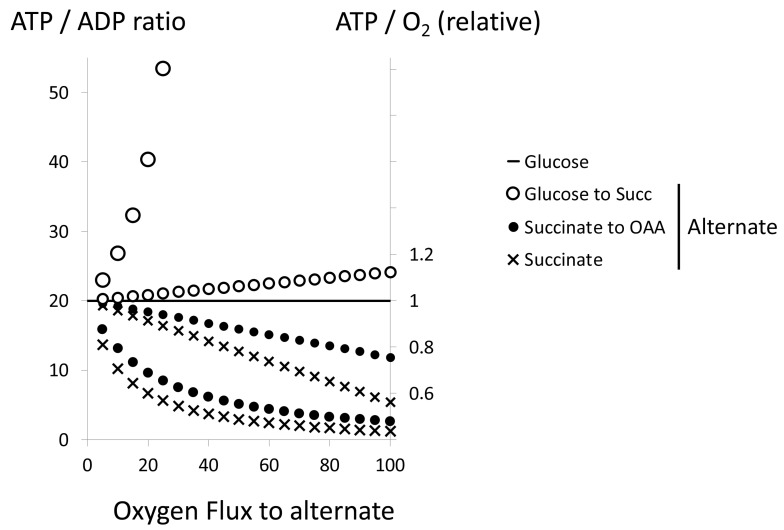
Succinate release or consumption. Model and calculation as in Figure 8. The succinate curve (SDH reaction) has the same values as in Figure 8 and Figure 9. Two other alternate pathways are considered: the partial oxidation of glucose into succinate (ATP/O_2_ = 6.4). The contribution of this pathway is predicted to increase ATP/ADP ratio abruptly, and calculation ends when all ADP is predicted to be converted into ATP (X ≥ 40). The complement for full oxidation of glucose (succinate to oxaloacetate) is then considered separately. Smaller symbols and right Y axis figure the relative changes in the ATP/O_2_ with regard to the reference value with glucose full oxidation (absolute value is 5.7), which highlights how minor variations in ATP/O_2_ considerably impact the ATP/ADP ratio.

However, there is an important difference between sulfide and succinate: sulfide toxicity makes its oxidation an absolute requirement to avoid poisoning in the short term, succinate does not show the toxicity of sulfide, and this is reflected by the relative affinities of the two enzymes for their respective substrates [27]. In the absence of succinate toxicity, incomplete glucose oxidation with succinate as the end product can be considered; it would maintain a significant mitochondrial ATP production rate with an ATP/O_2_ ratio of 6.4 [34]. In our model, it shows abrupt consequences for the ATP/ADP ratio (Figure 11). However, the relevant issue here is the twelve percent gain in oxygen efficiency. Succinate release appears therefore as an early marker for oxygen limitation and appropriate to convey the hypoxic message before threatening hypoxia would develop. Further adaptation to hypoxia could lead to anaerobic mode in the respiratory chain: complex II works backwards to re-oxidize quinone and then fumarate replaces oxygen as the final electron acceptor with the release of succinate. This adaptation, known for a long time in invertebrates [35], explains intense succinate accumulation during ischemia in mammals [36]. If high succinate concentrations stimulate vigorously complex II, it enters into fierce competition with complex I for the reduction of quinone (Figure 5) and may even drive electrons backwards into complex I [36], a situation similar to that observed with sulfide in colonocytes [19]. Then, the ATP/O_2_ tends toward the value of succinate oxidation, with consequences for the ATP/ADP ratio of the same order of magnitude or stronger than with sulfide.

## 5. Consequences of and Adaptations to Limitation in ATP Regeneration Rate

### 5.1. Timecourse and Cellular Content in Adenine Nucleotides

The intracellular content in adenine nucleotides appears pertinent in this model. The delay for the same interference between variation in mitochondrial bioenergetic and ATP/ADP ratio is dependent on the ratio between cellular adenine nucleotides (ATP, ADP, AMP) content and turnover. A lower concentration of adenine nucleotides implies a fast response, and oppositely, a high concentration (or a reserve of phosphocreatine) would average the influence of short-term variations in oxygen supply and/or mitochondrial bioenergetics.

### 5.2. Adaptive Response: Decrease in Cellular ATP Turnover and Its Consequences

There are two adaptive responses to a decrease in the ATP generation rate: (i) a decrease in ATP consumption rate and (ii) an increase in the capacity of the ATP generating machinery. The latter takes place in the mid-/long-term with the formation of new capillaries and/or improvement of mitochondrial fitness. In this respect, an increased endogenous sulfide tone compensated by increased SQR oxidation rate would be expected to drive adaptations related to that known for exercise and to improve physiological fitness.

In the short term, in order to avoid a continuous decrease in ATP/ADP ratio, the only option is to decrease the ATP hydrolysis rate, hence cellular energy expenditure. This has been extensively studied in animals needing to spare energy for short periods (diving) or in the long term (drought or cold). It requires a deeply depressed metabolic state for the whole animal or only for non-essential organs [37,38]. Notably, sulfide inhalation was able to induce such a hypometabolic state in mice [39]. It should be highlighted that in this study, two factors were applied simultaneously: sulfide inhalation and a decrease in ambient temperature. A reasonable hypothesis is that sulfide greatly enhances the adaptive response to adverse conditions, such as low temperature or hypoxia [40,41] resulting into entry of mice into torpor, a well-known adaptation to spare metabolic energy in small homeotherms, which is under neural (hypothalamic) control. In the absence of induction of “hibernation”, two factors are predicted to cause an automatic decrease in the activity of ATP hydrolyzing enzymes: (i) the decrease in ATP/ADP ratio would reduce the driving force for ATP hydrolysis but would jeopardize cellular viability. (ii) In contrast, a decrease in the cellular content of adenine nucleotides [42] would impact ATP consuming steps according to their affinity for ATP, which could reflect their priority.

Ionic balance is a major contributor to cellular energy expenditure; it results from the opposed activity of ionic pumps and channels. Ionic pumps establish a steady state for ion distribution across membranes that reflects the ATP/ADP ratio and channels generate a resting potential reflecting the repartition of the more permeant ion(s), which in the resting state is usually potassium. For this reason, impairment of oxidative phosphorylation, such as in mitochondrial intoxication or diseases, severely impacts neural function. A known mechanism is excitotoxicity, in which the primary event is the neurons’ failure to restore ionic balances after excitation. Closure of ionic channels is therefore a possible response to ATP shortage. Notably, in humans, the same mitochondrial mutation may have extreme deleterious consequences or alternatively may result in a persistent depolarization of plasma membrane with a more limited impact on patients [43]. The consequences of sulfide presence or oxidation for cellular bioenergetics could impact the polarization state of membranes and excitability, as has been observed with oxygen sensing cells [44]. This conclusion is pertinent with regard to the initial report on the possible role of sulfide as an endogenous neuromodulator [7]. In addition, simple models predict how the cellular load in sulfide (sum H_2_S + HS^−^) could be influenced by properties of the plasma membrane (Supplementary Figure S11).

### 5.3. Sustained Sulfide Oxidation Recalls the Effect of Mitochondrial Poisoning

Once a new steady state with sustained sulfide oxidation (and lower ATP turnover rate) would be attained, the cellular redox state would be affected. The priority for sulfide oxidation increases the reduction state of quinone and represses complex I; hence, it causes an increase in the NADH/NAD ratio. Moreover, lower complexes III/IV activities because of enzymatic limitation or oxygen shortage contribute to over reduction of the respiratory chain. Then, although cellular adaptations may ensure a new viable steady state, the consequences with regard to cellular redox state and ATP content would recall that of respiratory chain poisoning by an inhibitor like antimycin or cyanide.

## 6. Sulfide Oxidation Entails Two Oxygen Dependent Steps with Different Affinities

When strongly stimulated by sulfide concentration, the sulfide oxidation pathway shows a dependence on oxygen concentration in the 0–100 µM oxygen range [29] (Figure 7). This is likely to be explained by the dioxygenase reaction, whose affinity for oxygen was estimated with a Km of 25 µM [45], hence more than one order of magnitude higher than that of complex IV, which is lower than 1 µM [46]. Dissociation of oxygen from hemoglobin starts in the 50 µM range, transfer from capillaries to tissue implies a diffusion barrier with estimation of oxygen concentration outside capillaries of 16 mmHg (≈20 µM) [47] and finally oxygen concentration would be in the low micromolar range at the level of mitochondria. Therefore, because of the low affinity of the dioxygenase, sulfide oxidation would be much more affected by the variation of oxygen concentration in the physiological range than mitochondrial oxidation of other substrates. However, the latter is to be affected in a short time because imbalance between sulfide release and elimination initiates a self-amplified process leading to poisoning of cellular respiration by sulfide [29].

With the exception of gut epithelium, the maximal cellular/mitochondria sulfide oxidation capacity appears orders of magnitude higher than expected sulfide generating fluxes [19,30]. A possible explanation was the need to control the steady state concentration of sulfide at levels much lower than the Km of SQR for sulfide [30]. A similar reasoning may apply to dioxygenase and consequently to the control by oxygen concentration of the sulfide oxidation rate. Accordingly, the expression level of dioxygenase would determine the oxygen concentration at which sulfide oxidation balances a given sulfide releasing rate. Then, variations in dioxygenase expression level (activity) and sulfide release at the mitochondrial level would fine-tune a tolerable oxygen level below which sulfide would accumulate and within the short term, result in the inhibition of cellular respiration and major impairment of cellular bioenergetics.

## 7. Conclusions

Studies on cellular sulfide oxidation revealed its lowest ATP/O_2_ ratio when compared to other mitochondrial substrates. If respiring cells are exposed to an incoming flux of sulfide, the high affinity of SQR forces sulfide oxidation by mitochondria. This causes a large increase in cellular oxygen consumption because of the action of the dioxygenase acting downstream SQR to release oxidized sulfur ions. Moreover, interpretation of experiments suggests that a supplement in oxygen is made necessary because of an increase in electron transfer in the mitochondrial respiratory chain, which appears as a cellular adaptive response aiming to compensate for the influence of sulfide on the global ATP/O_2_.

A consequence would be that variations in the oxidation rates of low ATP/O_2_ ratio substrates greatly enhance cellular sensitivity to limitation of their mitochondrial bioenergetics by oxygen supply or by activity of the mitochondrial respiratory chain. If oxygen is the limiting factor, sulfide is by far the more influent. If limitation resides in the activity of the respiratory chain, sulfide is challenged by substrates whose oxidation is not dependent on NAD but involves FAD/FMN and direct reduction of quinone, such as succinate. The number of possible consequences caused by disturbances in cellular bioenergetics appears immense. However, in the first instance, the effects of sulfide donors, as well as mechanisms for oxygen sensing [44,48,49,50], may benefit from consideration of these interferences that take place even if variations in the fluxes of these critical substrates remain of marginal quantitative importance.

## Supplementary Materials

The following supporting information can be downloaded at: https://www.mdpi.com/article/10.3390/biom12030361/s1, Figure S1: Mitochondrial energization by nM sulfide; Figure S2: Impact of sulfide oxidation under oxygen limitation; Figure S3: Colored version of Figure 3; Figure S4: Colored version of Figure 4; Figure S5: Colored version of Figure 5; Figure S6: Colored version of Figure 6; Figure S7: Colored version of Figure 7; Figure S8: Colored version of Figure 8; Figure S9: Colored version of Figure 9; Figure S10: Colored version of Figure 10; Figure S11: Model for sulfide permeation and cellular concentration.
