# Supplementary material for: Sulfide Oxidation Evidences the Immediate Cellular Response to a Decrease in the Mitochondrial ATP/O2 Ratio
Source: Biomolecules. 2022 Feb 24;12(3):361. doi: 10.3390/biom12030361 (PMC8944965; doi:10.3390/biom12030361)
Supplement: Supplementary file 1 [file biomolecules-12-00361-s001.zip › biomolecules-1568843-supplementary.pdf]

Cellular sulfide oxidation causes decrease in ATP/O_2 ratio with immediate bioenergetic response.

F Bouillaud

Biomolecules 2022

Supplementary Figures

Supplementary Figure S1: Mitochondrial energization by nM sulfide

Supplementary Figure S2: Impact of sulfide oxidation under oxygen limitation

Supplementary Figure S3: Colored version of Figure 3

Supplementary Figure S4: Colored version of Figure 4

Supplementary Figure S5: Colored version of Figure 5

Supplementary Figure S6: Colored version of Figure 6

Supplementary Figure S7: Colored version of Figure 7

Supplementary Figure S8: Colored version of Figure 8

Supplementary Figure S9: Colored version of Figure 9

Supplementary Figure S10: Colored version of Figure 10

Supplementary Figure S11: Model for sulfide permeation and cellular concentration

Supplementary Figure S1: Mitochondrial energization by nM sulfide

Flow cytometry: Liver biopsy homogenate,

Y axis: Green fluorescence (membrane potential)

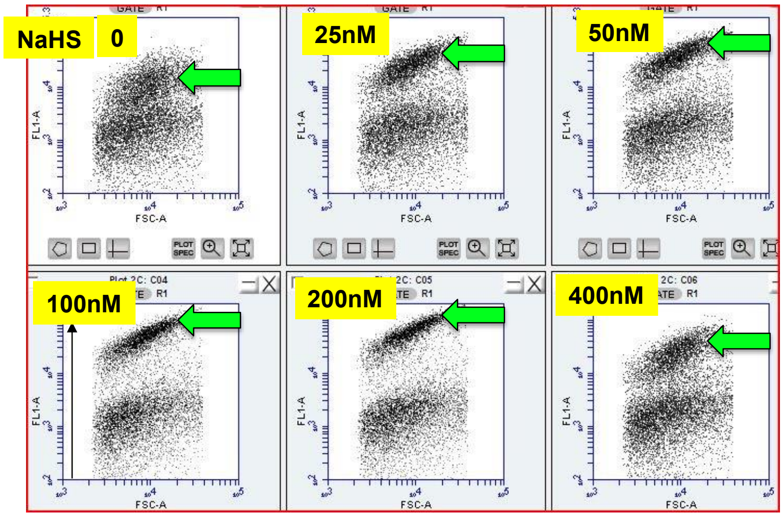

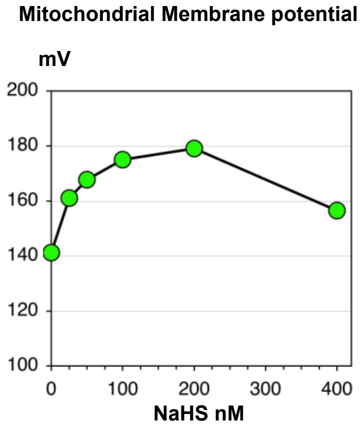

Glut / Mal 5 mM	170 mV
Succinate 7.5mM	172 mV
NaHS max	184 mV

X axis: FSC (size)

population of objects with a membrane potential (mitochondria)

Legend:
C. Prip-Buus, F. Bouillaud (2012) Collaboration with: N Helmy, C. Vons, (Jean Verdier Hospital Bondy France).
Homogenate from a human liver biopsy was incubated in mitochondrial respiration medium in absence of ADP and substrates. Mitochondrial membrane potential was evaluated according to the accumulation of the membrane potential probe Rhodamine 1,2,3. Increasing concentrations (0,25, 50, 100, 200, 400 nM) of sodium hemisulfide (NaHS) were added to different samples and reading was performed immediately with a flow cytometer Accuri C6, detection of objects was made according to forward scatter (FSC). Mitochondria are recognized by their high fluorescence (green arrow). Estimation of mitochondrial membrane potential is given on the right and compared with the values obtained with the same homogenate and usual mitochondrial substrates. With this procedure it could be evidenced that sulfide caused an increase in membrane potential in the range 25-200nM, which could be attributed to the activity of SQR. 400nM caused depolarization when compared to 200nM. This is likely to reflect that this concentration of sulfide is above the Ki (value 0.2µM in reference 2) for inhibition of complex IV by sulfide.
Two parameters explain the high sensitivity of this procedure to evidence SQR activity with these low concentrations of sulfide: 1) the extreme sensitivity of membrane potential to minor increase in electron transfer in the respiratory chain in absence of ADP. 2) The high dilution of mitochondria, which allow the low amount of sulfide added to sustain mitochondrial respiration for a significant period of time before being exhausted.
Mat & Methods One preparation from those presented in Helmy et al. 2014, (ref 29). For more details see this reference and for flow cytometry procedures and calculation of membrane potential see: M. Damiano et al., "Tissue- and cell-specific mitochondrial defect in Parkin-deficient mice," PLoS One, vol. 9, no. 6, p. e99898, 2014, doi: 10.1371/journal.pone.0099898

Supplementary Figure S2: Impact of sulfide oxidation under oxygen limitation

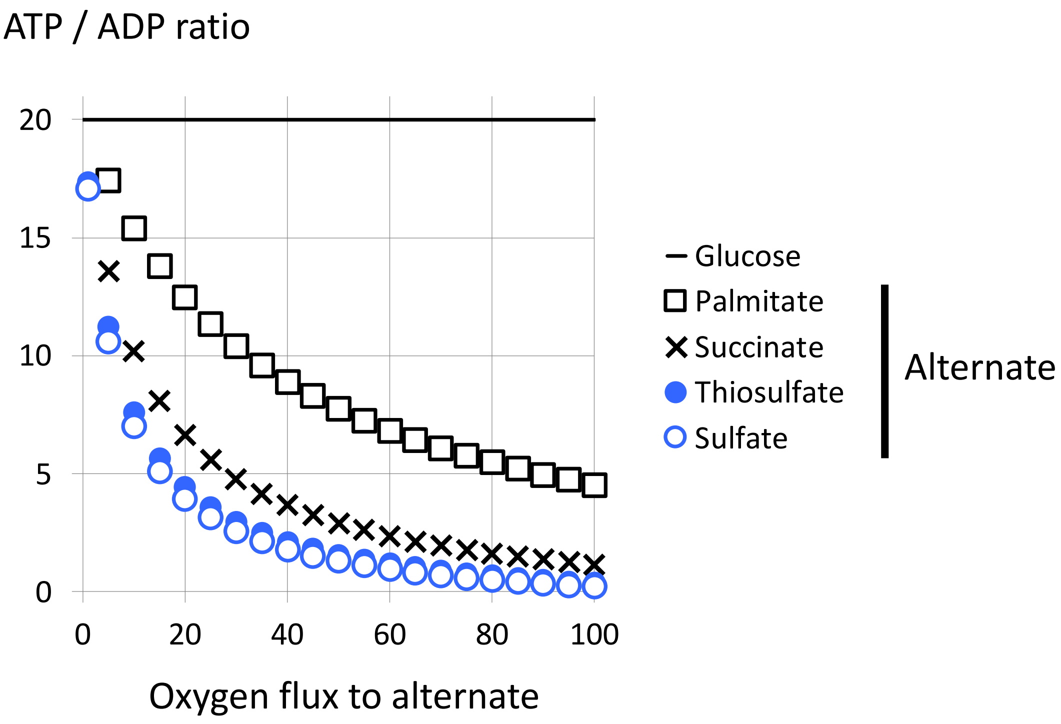

Legend: Same as in Figure 9 except that the X axis represent oxygen diverted to the alternate substrate in all cases. Then the difference depends only on the ATP/O_2 ratio and results in almost identical consequences for the oxidation in sulfate (ATP/O2=1.35) or thiosulfate (ATP/O2=1.6), other values are 3.2 for SDH/complex II reaction, 4.9 for palmitate full oxidation and 5.7 for the reference: full oxidation of glucose.

Supplementary Figure S3: Colored version of Figure 3

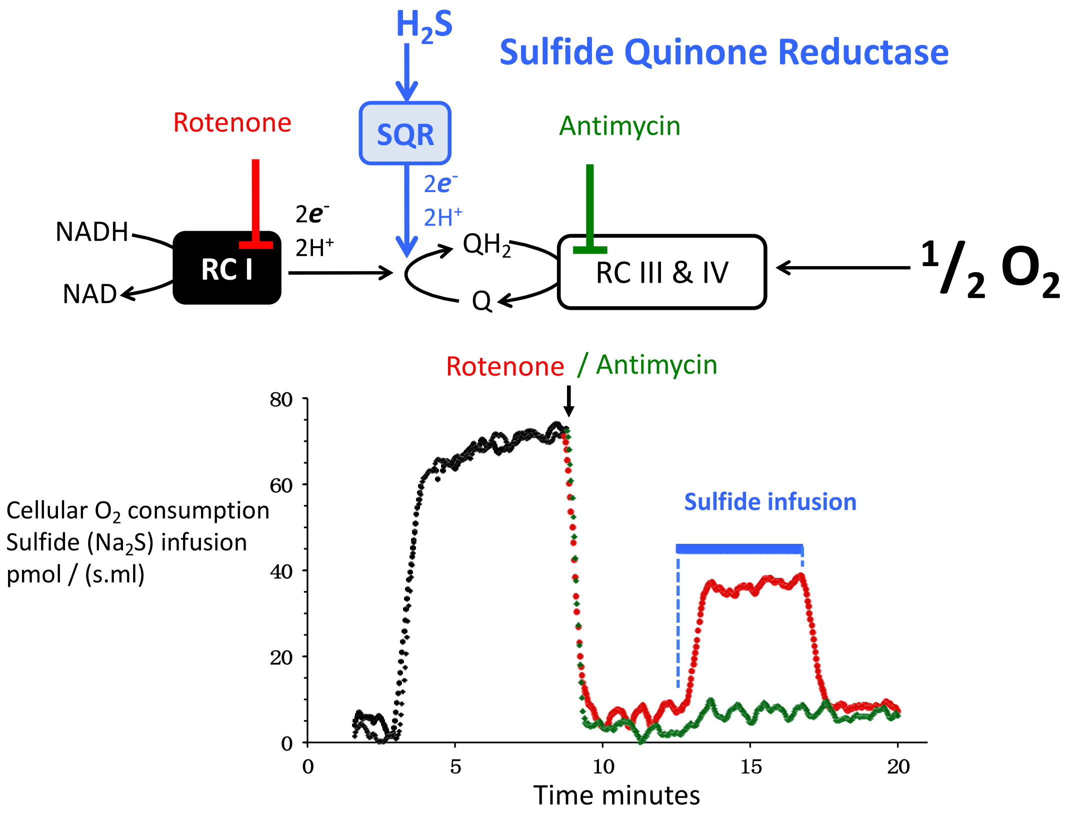

Legend: Note that the actual number of experimental data points (each two seconds) is represented here, one out of four was shown in figure 3 for the sake of clarity.

Supplementary Figure S4: Colored version of Figure 4

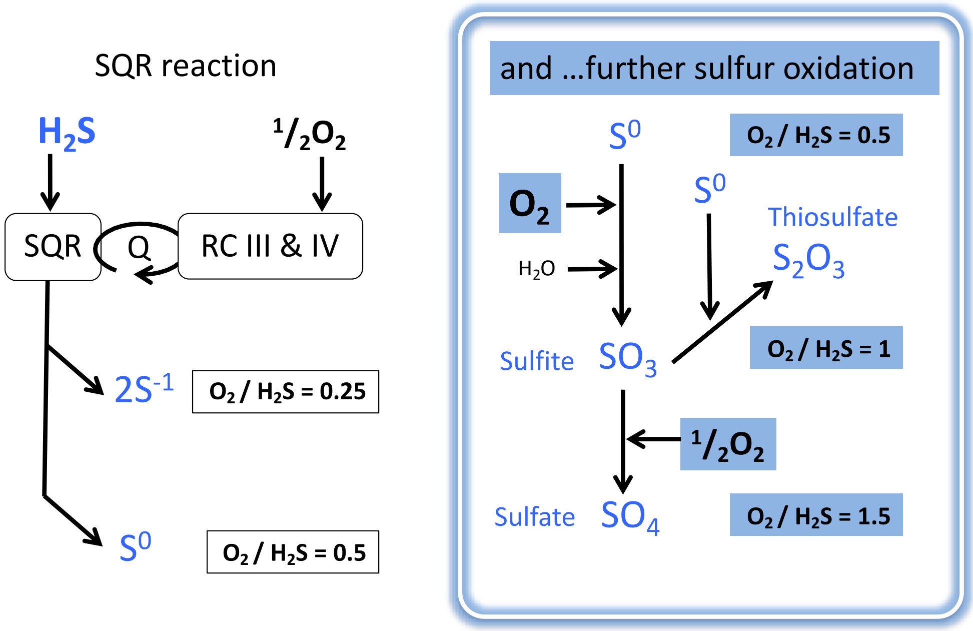

Supplementary Figure S5: *Colored version of Figure 5*

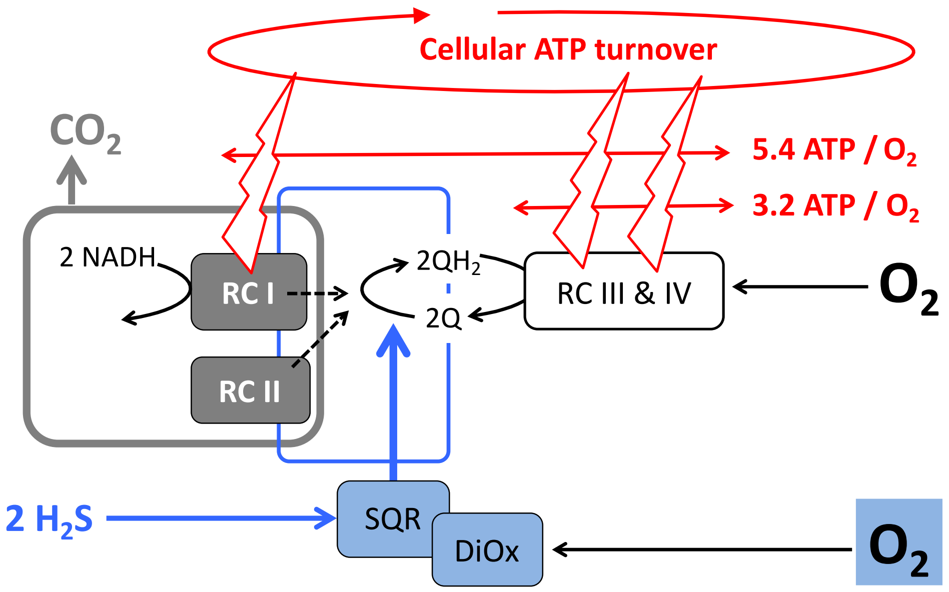

Supplementary Figure S6: *Colored version of Figure 6*

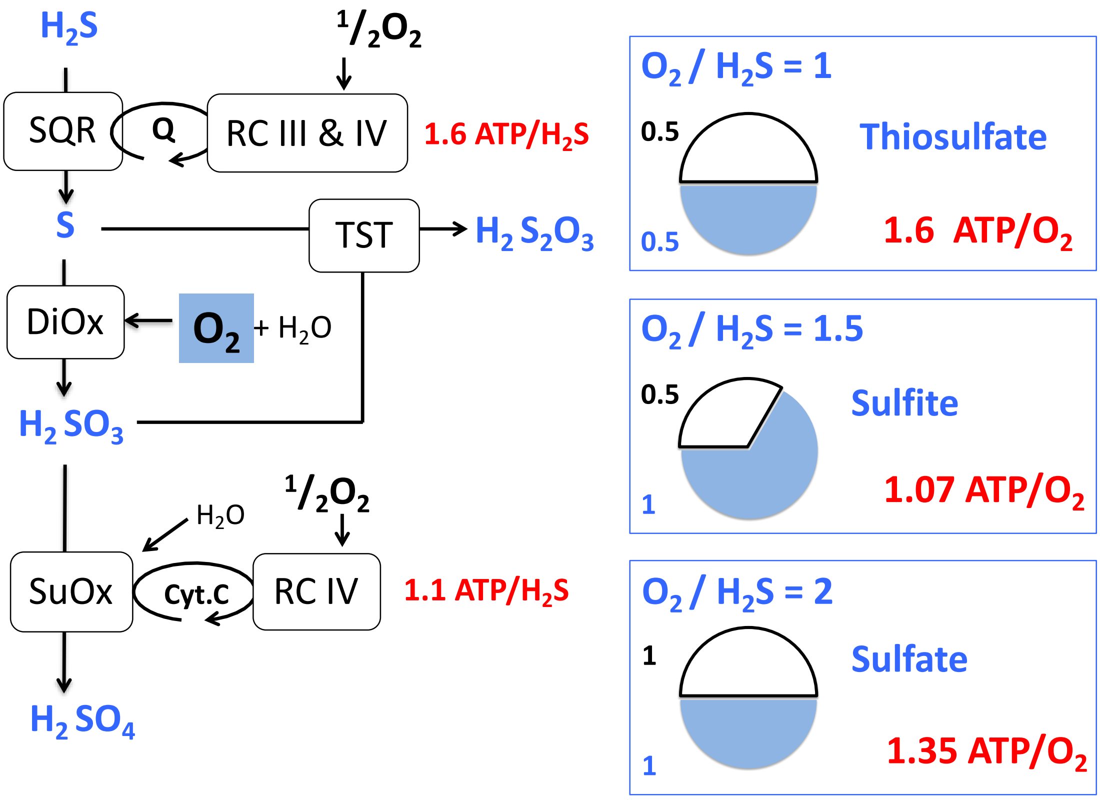

Supplementary Figure S7: Colored version of Figure 7

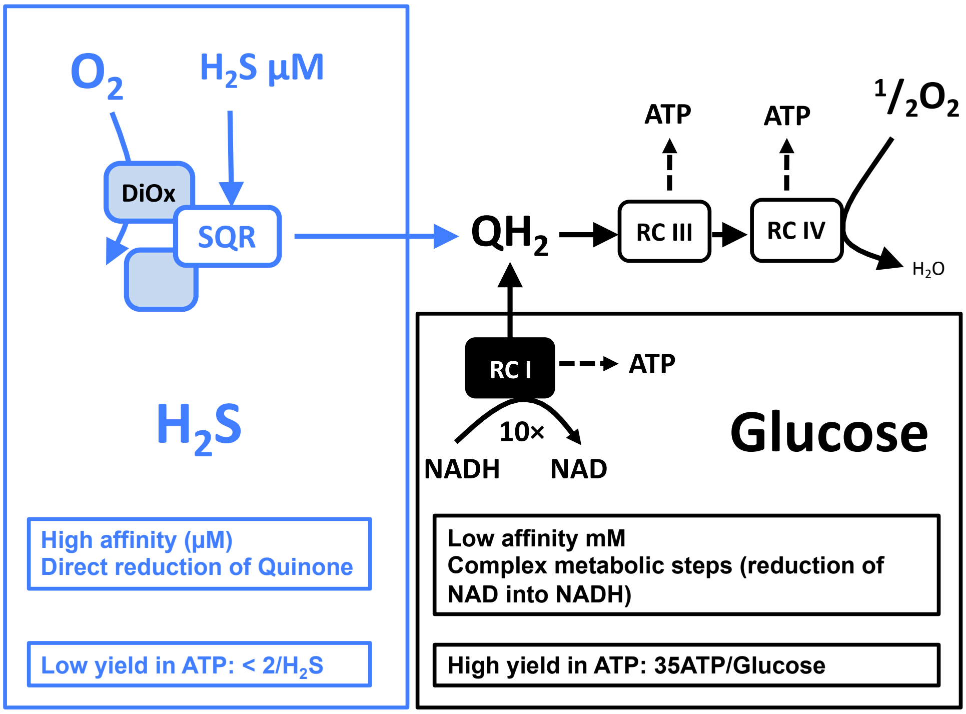

Supplementary Figure S8: Colored version of Figure 8

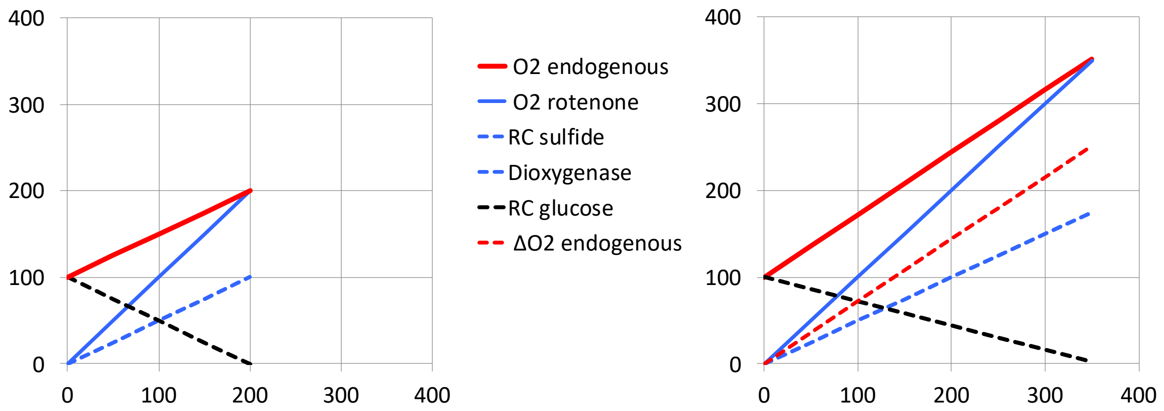

Supplementary Figure S9:   Colored version of Figure 9

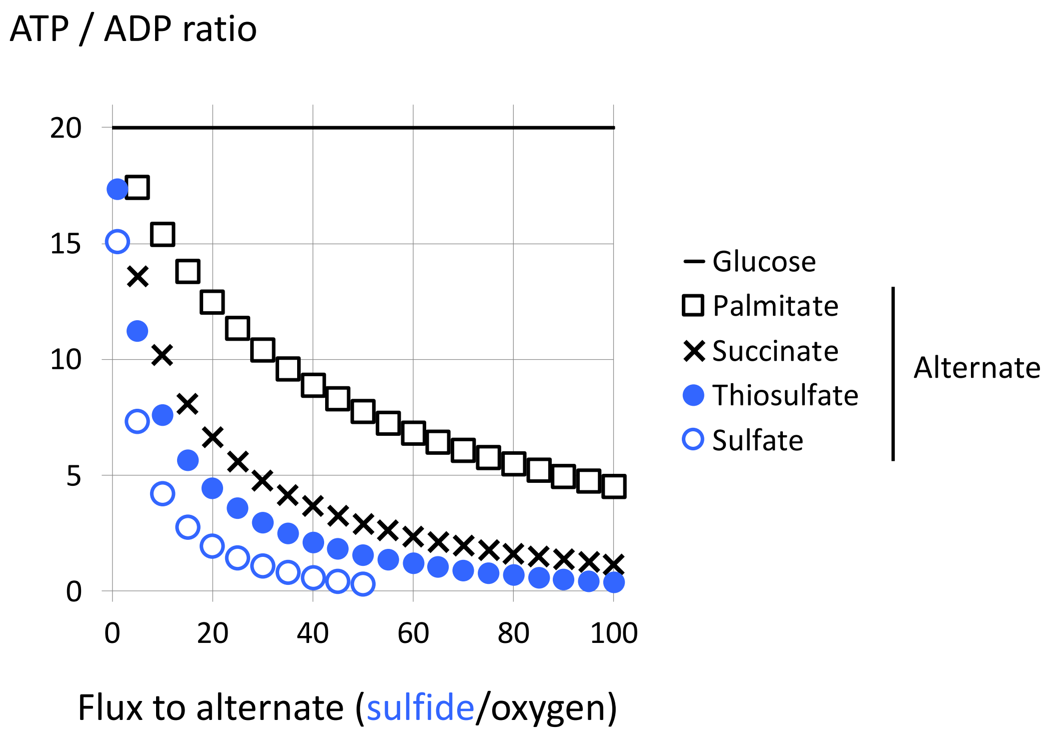

Supplementary Figure S10: Colored version of Figure 10

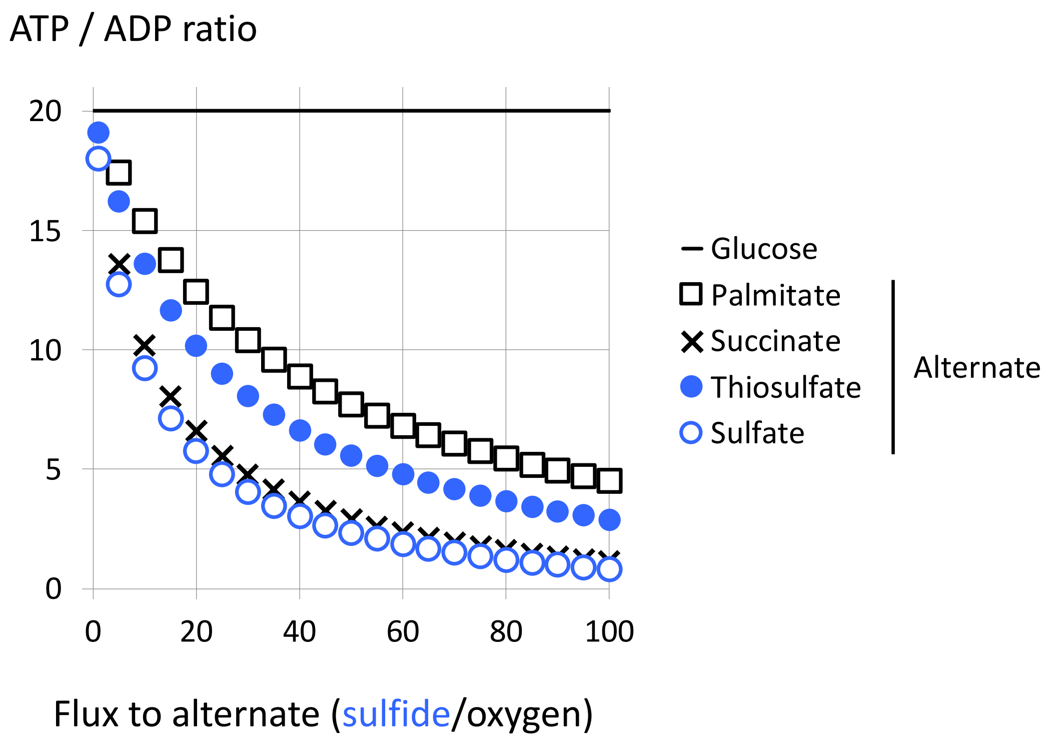

Supplementary Figure S11: Model for sulfide permeation and cellular concentration

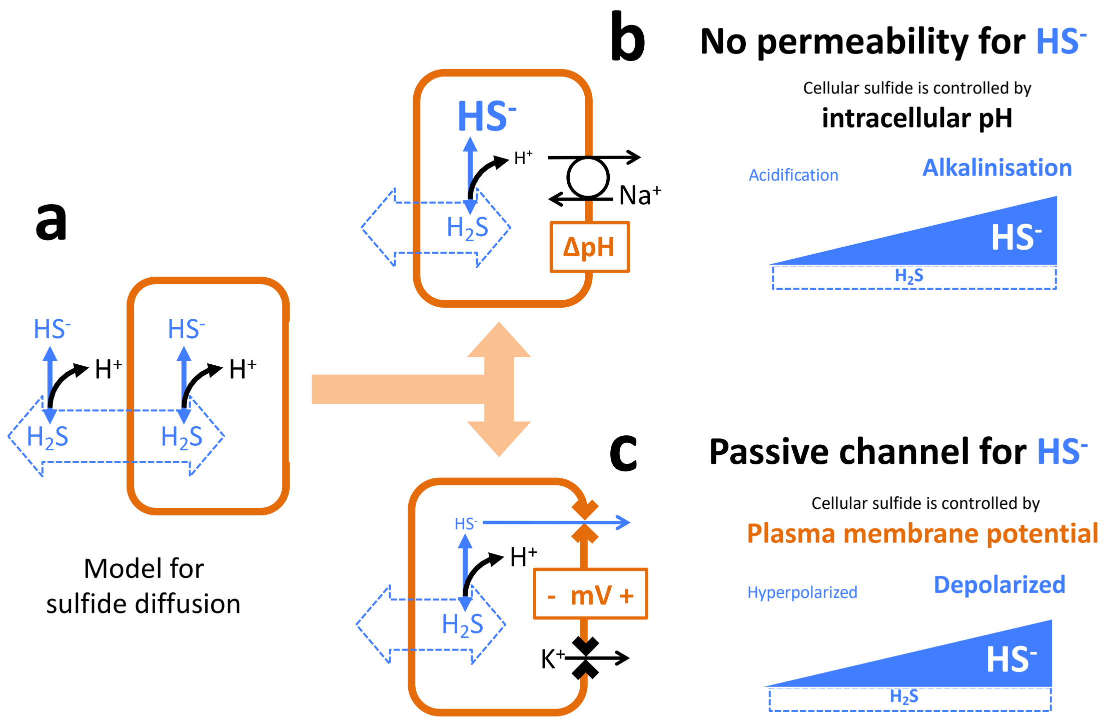

Legend:

a: *model for sulfide permeation in/out of a cell. The sulfide content is the sum of H_2S and hemisulfide (HS^- anion) because sulfide ion (S^{2-}) is negligible at physiological pH. The ratio between H_2S and hemisulfide is determined by the pH of the solution. H_2S (gas) is permeant across the lipidic membrane. It results an equilibration of H_2S concentration between outside and inside (dotted, two-sided arrow).*

b: *if plasma membrane is impermeable to hemisulfide, its intracellular concentration is determined by the value of intracellular pH and increases with cellular alkalinization, represented here to result from enhanced proton sodium exchange.*

c: *If a passive conductance (channel) exists for hemisulfide, then the plasma membrane potential (negative inside) drives it out of the cell and consequently hyperpolarization lowers cellular sulfide concentration. Plasma membrane resting potential results from activity of potassium channel(s). If cellular sulfide causes directly or indirectly inhibition of potassium channel(s), a transient cellular depolarization event would be stabilized by the increase in intracellular sulfide it causes, which may appear as a primitive form of memory.*
